# Laparoscopic Evaluation and the Management of the Nonpalpable Testis

**DOI:** 10.1155/DTE.4.69

**Published:** 1997

**Authors:** Toshiki Koyama, Katsuya Nonomura, Kaname Ameda, Hidehiro Kakizaki, Yasukuni Matsugase, Yuichiro Shinno, Takayuki Kanno, Tetsufumi Yamashita, Masashi Murakumo, Tomohiko Koyanagi

**Affiliations:** Department of Urology Hokkaido University School of Medicine North-15, West-7 Kita-ku Sapporo city 060 Japan

## Abstract

From June 1992 to December 1996, we performed laparoscopic evaluation for 28 nonpalpable testes in 22 patients (1–21, median 3 years old).

The location of 28 testes were divided into 4 categories according to the classification by Malone *et al*.: canalicular in 17 testes, just canalicular in 2, abdominal in 7, and absent in 2. Two-stage Fowler–Stephens orchiopexy was performed in 3 abdominal testes and planned two-stage orchiopexy was performed in one abdominal testis, while one-stage standard orchiopexy was performed in 10 testes (canalicular 5, just canalicular 2, and abdominal 3). In 10 of 17 canalicular testes no testicular element was found on histological examination of the excised remnant tissue. In two completely absent testicular structures, as verified by vanishing spermatic vessels, no further exploration was done after laparoscopy. There was one complication in this series: jejunal injury which needed oversewing, otherwise there was no postoperative sequela in all cases.

Laparoscopic evaluation in patients with nonpalpable testes gives us precise information as to the existence and location of the testicle which is helpful in determining subsequent appropriate procedure and avoiding unnecessary abdominal exploration.


*Diagnostic and Therapeutic Endoscopy*, Vol. 4, pp. 69–74
Reprints available directly from the publisher
Photocopying permitted by license only

© 1997 OPA (Overseas Publishers Association)
Amsterdam B.V. Published under license
in The Netherlands under the Harwood Academic
Publishers imprint, part of The Gordon and
Breach Publishing Group.
Printed in Singapore.

### Laparoscopic Evaluation and the Management of the Nonpalpable Testis

TOSHIKI KOYAMA*, KATSUYA NONOMURA, KANAME AMEDA, HIDEHIRO KAKIZAKI, YASUKUNI MATSUGASE, YUICHIRO SHINNO, TAKAYUKI KANNO, TETSUFUMI YAMASHITA, MASASHI MURAKUMO and TOMOHIKO KOYANAGI

*Department of Urology, Hokkaido University School of Medicine, North-15, West-7, Kita-ku, Sapporo city 060, Japan*

*(Received 3 April 1997; In final form 13 May 1997)*

**From June 1992 to December 1996, we performed laparoscopic evaluation for 28 nonpalpable testes in 22 patients (1–21, median 3 years old).**

**The location of 28 testes were divided into 4 categories according to the classification by Malone *et al.*: canalicular in 17 testes, just canalicular in 2, abdominal in 7, and absent in 2. Two-stage Fowler–Stephens orchiopexy was performed in 3 abdominal testes and planned two-stage orchiopexy was performed in one abdominal testis, while one-stage standard orchiopexy was performed in 10 testes (canalicular 5, just canalicular 2, and abdominal 3). In 10 of 17 canalicular testes no testicular element was found on histological examination of the excised remnant tissue. In two completely absent testicular structures, as verified by vanishing spermatic vessels, no further exploration was done after laparoscopy. There was one complication in this series: jejunal injury which needed oversewing, otherwise there was no postoperative sequela in all cases.**

**Laparoscopic evaluation in patients with nonpalpable testes gives us precise information as to the existence and location of the testicle which is helpful in determining subsequent appropriate procedure and avoiding unnecessary abdominal exploration.**

*Keywords:* Cryptorchidism, Inguinal exploration, Laparoscopy, Nonpalpable testis

### INTRODUCTION

Nonpalpable testes account for 13–43% of all cases of cryptorchidism, the average being 20% [1]. Many reports on the use of laparoscopy in nonpalpable testes have been published, and it has been demonstrated to be the most reliable, safe and effective procedure in the evaluation of nonpalpable testes [2–11]. The principal goal of laparoscopy in nonpalpable testes is to provide information regarding testicular presence and location to facilitate the most appropriate surgical approach [8]. Accurate laparoscopic assessment and localization of testis could obviate further surgical exploration in cases of testicular absence or vanishing testis syndrome [9]. Herein, we describe

*Corresponding author. Tel.: +81-11-716-1161 ext. 5949. Fax: +81-11-736-8052.

our experience to clarify the usefulness of laparoscopy in the diagnosis and management of nonpalpable testes.

### MATERIALS AND METHODS

Between June 1992 and December 1996, 28 nonpalpable testes in 22 patients were evaluated by laparoscopy. Patient age ranged from 1 to 21 (median 3) years old. Six patients had bilateral nonpalpable testes and 16 patients had unilateral nonpalpable testis. Of the unilateral nonpalpable testes, 11 were on the left side and 5 were on the right side. Laparoscopy was indicated when a testis was physically nonpalpable and not detected on ultrasonographic examination in the inguinal region.

Laparoscopy was performed under general anesthesia. Each patient was reexamined whether the testes were palpable after induction of general anesthesia. During the laparoscopic procedure the bladder drainage by urethral catheterization was the rule. In the initial 3 cases, we used a Veress needle to insufflate the abdomen, but now this had been changed to an open method for avoiding an insufficient insufflation and penetration of a viscus or major blood vessels. A small infraumbilical skin incision was made and peritoneum was opened. Through this incision a 5 or 10 mm sized trocar was certainly and safely introduced into the peritoneal cavity and pneumoperitoneum was created using carbon dioxide with about 8 mmHg of intra-abdominal pressure. The telescope system was inserted into the peritoneal cavity via the trocar. The patient was postured in the Trendelenburg position in most of the cases.

The internal ring on the affected side was found first through the laparoscope. In accordance with the classification by Malone and Guiney [3], we divided 28 nonpalpable testes into 4 categories (Fig. 1).

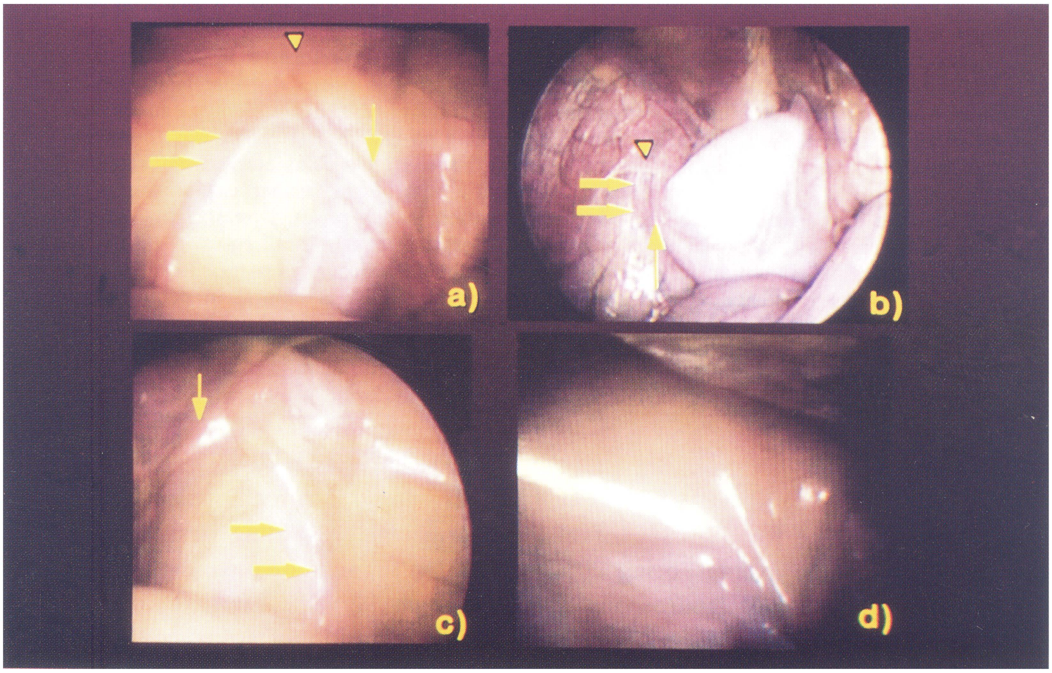

FIGURE 1 Laparoscopic findings of nonpalpable testes: (a) canalicular (left side), (b) just canalicular (left side), (c) abdominal (right side) and (d) absent (left side) (arrowhead indicates the internal inguinal ring; thick arrow, gonadal vessels; and thin arrow, the vas deferens).

(a) *Canalicular*: Normal-sized vas deferens and gonadal vessels are seen entering the canal through the internal ring. The testis is not visible and its existence is uncertain.
(b) *Just canalicular*: The testis lies with its lower pole just touching or partially entering the internal ring. Vas and vessels are seen.
(c) *Abdominal*: The testis lies clearly within the abdomen at a distance usually of 1–3 cm from the internal ring.
(d) *Absent or atrophic*: A normal vas is seen but the vessels are either absent or visibly hypoplastic.

After establishing the diagnosis by laparoscopy, a therapeutic operation such as open inguinal orchiopexy or laparoscopic surgery is performed in the same session.

At the laparoscopic surgical manipulation, two additional trocars were inserted and dissecting forceps and end clips were introduced via these trocars.

### RESULTS

Laparoscopy was technically successful in all of the 28 nonpalpable testes. Location of the 28 nonpalpable testes and subsequent managements are shown in Table I. Of all 28 nonpalpable testes, 17 were canalicular testes. In all these canalicular testes, exploration of the inguinal canal were performed. Five were fixed to dartos pouch of the ipsilateral scrotum as a standard manner and the other 12 absolutely small testes and testicular nubbins were removed. Histological examination of these 12 testicular nubbins revealed structure of seminiferous tubules in only 2 specimens and no testicular tissue in 10 nubbins. Internal inguinal ring was closed in 16 cases of these canalicular testes and internal inguinal ring and processus vaginalis was patent only in 1 case. In 2 'just canalicular' testes internal inguinal ring was also patent and standard orchiopexy was successfully performed. In our series 7 testes were located in the abdominal cavity. In 3 of these abdominal testes, standard orchiopexy could be performed. In one abdominal testis, laparoscopy demonstrated that the testicular vessels were too short to pull out the testis in scrotum. No further open exploration was done at that time for planning the testicular autotransplantation using microvascular technique later [12]. The testicular vessels were resultantly long enough to perform standard orchiopexy at the staged operation in this case. Other 3 abdominal testes were also located far from the internal inguinal ring and their testicular vessels were apparently short so that the testicular vessels were clipped under laparoscopy as the first stage operation of two-stage Fowler–Stephens orchiopexy (Fig. 2). The patients successfully underwent the second stage operation of two-stage Fowler–Stephens orchiopexy 6 months later. Two were absent and no further open exploration was done.

There was one complication in this series. When the peritoneum was opened, the jejunum was inadvertently injured. The jejunum was needed oversewing and a nasogastric tube was placed for 5 days. There was no more complication and this patient discharged at 10th hospital day after the operation while other patients were hospitalized for several days after the operation.

TABLE I Location of testes and subsequent management

| Type | Orchiectomy | Orchiopexy | No surgery | Total |
|---|---|---|---|---|
| Canalicular | 12 | 5 | | 17 |
| Just canalicular | | 2 | | 2 |
| Abdominal | | 6* | 1† | 7 |
| Absent | | | 2 | 2 |
| Total | 12 | 13 | 3 | 28 |

*First stage of Fowler–Stephens orchiopexy was performed in 3 cases.
† Standard orchiopexy was performed 12 months later.

### DISCUSSION

Nonpalpable testis requires several aspects of diagnostic and therapeutic consideration. Many methods for detection of the nonpalpable testis have been described, including ultrasonography

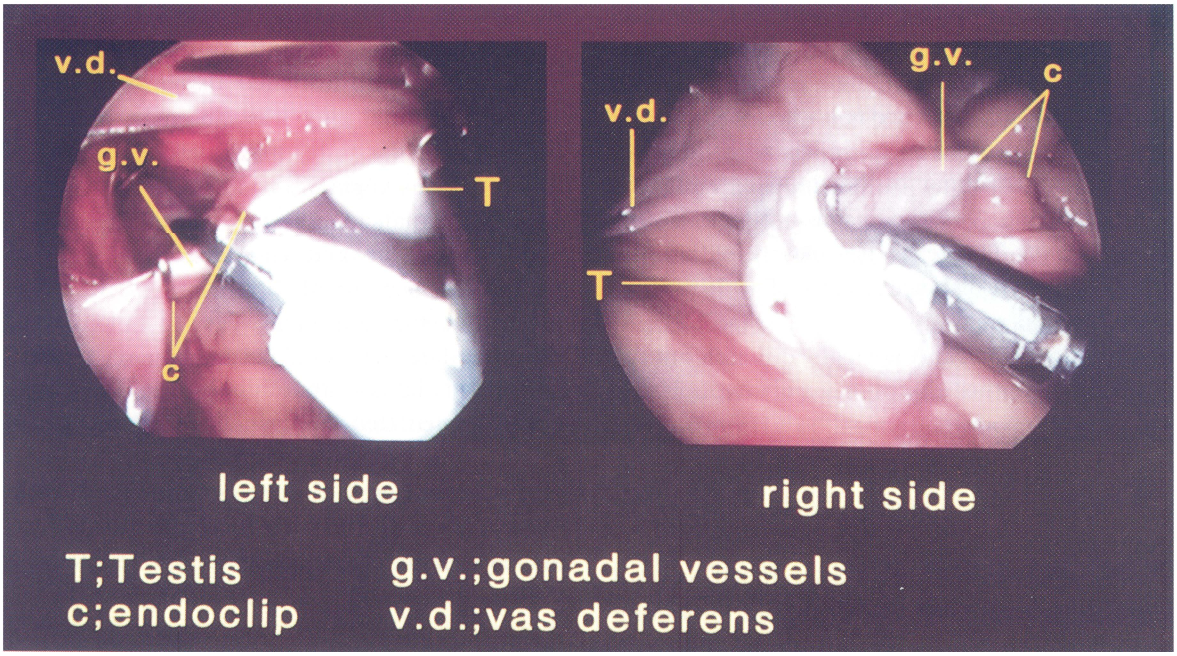

FIGURE 2 The case of bilateral abdominal testes. First stage of Fowler–Stephens orchiopexy was completed (gonadal vessels were clipped under laparoscope).

(US), computed tomography (CT), magnetic resonance imaging (MRI) and angiography [13–16] although none has been widely accepted as reliable examination. US avoids the use of ionizing radiation, permits evaluation without sedation, and is not expensive. However, it can identify only larger testes located in the inguinal canal which are at juxtavesical position. With CT, the testes are easily identified if they are located beneath the inguinal canal, but it emits radiation and it is difficult to perform in the young children because of long scanning time and the need for sedation. MRI may be useful to document the location of testis, but it is expensive and is also difficult to perform in the young children. Angiography is technically difficult and invasive procedures in the young children. All these imaging procedures are limiting and if the testes are not identified by these procedures, the exploration of the inguinal canal and abdominal cavity is resultantly necessary.

The laparoscopy, which only takes about 15 min, has the advantage of more accurate diagnostic modality for detection of dislocated testes or proving their absence through a fine-vision optical lens. Diagnostic laparoscopic procedure could also let us determine the subsequent appropriate management for the testes. Subsequent surgical management according to the laparoscopic findings can be decided as shown in Table II. Unnecessary surgical exploration can be avoided in absent cases, and alternative procedures can be selected in abdominal cases.

If the abdominal testis is found close to the internal inguinal ring and its testicular vessel is long enough, standard orchiopexy is indicated in the same session. If the testis is located far from the internal inguinal ring and its testicular vessel is short, another complicated procedure should be selected. In these cases, not only two-stage orchiopexy [17] but also one-stage [18] or two-stage Fowler–Stephens orchiopexy [19,20] and

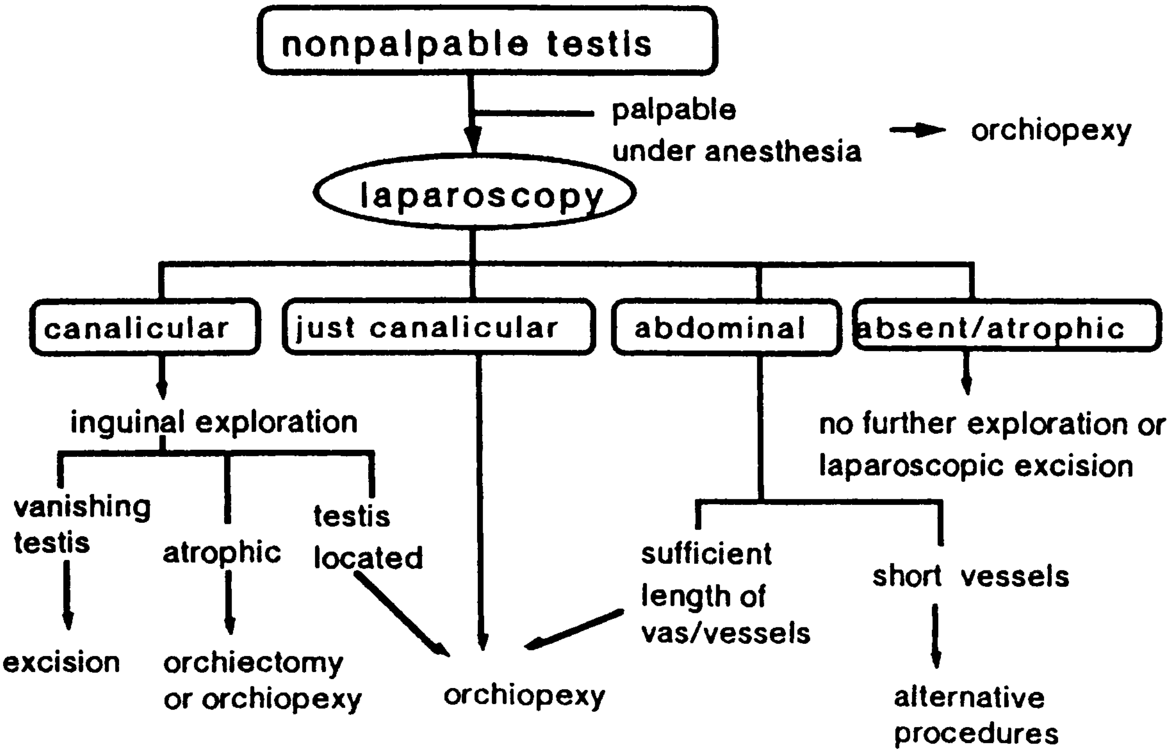

TABLE II Algorithm for management of nonpalpable testis autotransplant with microvascular technique [12] can be preferable to perform. Recently, therapeutic laparoscopic procedures have been applied to nonpalpable testes including laparoscopic orchiectomy, laparoscopic assisted orchiopexy and first stage and second stage of Fowler–Stephens orchiopexy [7,21–23]. So far, there is no universal opinion to account these additional procedures as standard technique for nonpalpable testes because they are more complicated and require long operation time.

In conclusion, laparoscopic evaluation in patients with nonpalpable testes gives us precise information as to the existence and location of the testicle, which is helpful in determining subsequent appropriate procedure and avoiding unnecessary abdominal exploration even in our small experience. Although laparoscopy is less invasive procedure, meticulous care must be taken to avoid inherent complications to the alimentary system.

### *References*

[1] Levitt, S.B., Kogan, S.J., Engel, R.M. *et al.* The impalpable testes: A rational approach to management. *J. Urol.* 1978; **120**: 515–520.

[2] Lowe, D.H., Brock, W.A. and Kaplan, G.W. Laparoscopy for nonpalpable testes. *J. Urol.* 1984; **131**: 728–729.

[3] Malone, P.S. and Guiney, E.J. The value of laparoscopy in localizing the impalpable undescended testis. *Br. J. Urol.* 1984; **56**: 429–431.

[4] Guiney, E.J., Corbally, M. and Malone, P.S. Laparoscopy and the management of the impalpable tesis. *Br. J. Urol.* 1989; **63**: 313–316.

[5] Castilho, L.N. Laparoscopy for the nonpalpable testis: How to interpret the endoscopic findings. *J. Urol.* 1990; **144**: 1215–1218.

[6] Wilson-Storey, D. and MacKinnon, A.E. The laparoscope and the undescended testis. *J. Pediatr. Surg.* 1992; **27**: 89–92.

[7] Poenaru, D., Homsy, Y.L., Peloquin, F. *et al.* Laparoscopic management of the impalpable testis. *Urology* 1993; **42**: 574–579.

[8] Moore, R.G., Peters, C.A., Bauer, S.B. *et al.* Laparoscopic evaluation of the nonpalpable testis: A prospective assessment of accuracy. *J. Urol.* 1994; **151**: 728–731.

[9] Tennenbaum, S.Y., Lerner, S.E., McAleer, L.M. *et al.* Preoperative laparoscopic locating of the nonpalpable testis: A critical analysis of a 10-year experience. *J. Urol.* 1994; **151**: 732–734.

[10] Elder, J.S. Laparoscopy for impalpable testes: Significance of the patent processus vaginalis. *J. Urol.* 1994; **152**: 776–778.

[11] Fukuzaki, A., Chiba, Y. and Orikasa, S. Laparoscopic evaluation and subsequent management of nonpalpable testis. *Jap. J. Endourol. ESWL* 1995; **8**: 130–133.

[12] Wacksman, J., Dinner, M. and Handler, M. Results of testicular autotransplantation using the microvascular technique: Experience with 8 intra-abdominal testes. *J. Urol.* 1982; **128**: 1319–1321.

[13] Malone, P.S. and Guiney, E.J. A comparison between ultrasonography and laparoscopy in localizing the impalpable undescended testes. *Br. J. Urol.* 1985; **57**: 185.

[14] Lee, J.K. and Glazer, H.S. Computed tomography in the localization of the nonpalpable testis. *Urol. Clin. North Am.* 1982; **9**: 397–404.

[15] Kier, R., McCarthy, S., Rosenfield, A.T. *et al.* Nonpalpable testes in young boys: Evaluation with MR imaging. *Radiology* 1988; **169**: 429.

[16] Ben-Menachen, Y., deBaradinis, M.C. and Salinas, R. Localization of intra-abdominal testes by selective testicular arteriography: A case report. *J. Urol.* 1974; **112**: 493–494.

[17] Steinhardt, G.F., Kroovand, R.L. and Perlmutter, A.D. Orchiopexy: Planned 2-stage technique. *J. Urol.* 1985; **133**: 434–435.

[18] Koff, S.A. and Sethi, P.S. Treatment of high undescended testes by low spermatic vessel ligation: An alternative to the Fowler–Stephens technique. *J. Urol.* 1996; **156**: 799–803.

[19] Ransley, P.G., Vordermark, J.S., Caldamone, A.A. *et al.* Preliminary ligation of the gonadal vessels prior to orchiopexy for the intra-abdominal testicle. A staged Fowler–Stephens procedure. *World J. Urol.* 1984; **2**: 266–268.

[20] Elder, J. Two-stage Fowler–Stephens orchiopexy in the management of intra-abdominal testes. *J. Urol.* 1992; **148**: 1239–1241.

[21] Bloom, D.A. Two-step orchiopexy with pelviscopic clip ligation of the spermatic vessels. *J. Urol.* 1991; **145**: 1030–1033.

[22] Jordan, G.H., Robey, E.L. and Winslow, B.H. Laparoscopic surgical management of the abdominal/transinguinal undescended testicle. *J. Endourol.* 1992; **6**: 159–163.

[23] Bogaert, G.A., Kogan, B.A. and Mevorach, R.A. Therapeutic laparoscopy for intra-abdominal testes. *Urology* 1993; **42**: 182–188.

